# Economic and organizational impact of oral versus intravenous and subcutaneous administration of hypomethylating agents in patients with acute myeloid leukemia not eligible for intensive chemotherapy in Spain

**DOI:** 10.1371/journal.pone.0358426

**Published:** 2026-09-22

**Authors:** María Lloret Avellá, Mafalda Carmo, Núria Paladio Duran, Pere Blancher Parcerisa

**Affiliations:** 1 IQVIA, Barcelona, Spain; 2 Otsuka, Barcelona, Spain; Stanford University, UNITED STATES OF AMERICA

## Abstract

Acute myeloid leukemia (AML) primarily affects older adults, many of whom are unsuitable for intensive chemotherapy. Hypomethylating agents (HMA) -intravenous (IV) decitabine and subcutaneous (SC) azacitidine- are standard therapeutic options but require frequent hospital visits and substantial healthcare resource use. Oral decitabine/cedazuridine (DEC-C) offers a therapeutically equivalent alternative that may reduce hospital resource burden and improve patient convenience. This study estimated and compared the economic and organizational impact of IV, SC, and oral HMA administration in AML patients unfit for standard intensive chemotherapy, from the perspective of the Spanish National Health System. A micro‑costing model quantified direct non‑pharmacological costs associated solely with the administration route across five domains: drug preparation and administration, treatment‑related hospitalizations, medical transportation, catheter management, and infections. Resource use was derived from a survey of 30 healthcare professionals across 16 hospitals and valued using official tariffs. Costs were calculated separately for induction and subsequent cycles; drug acquisition costs were excluded. Non‑pharmacological costs per patient were €9,998 for IV decitabine, €12,177 for SC azacitidine, and €1,306 for oral DEC‑C, representing cost reductions of 87% and 89% for oral therapy versus IV and SC, respectively. Savings were driven mainly by reductions in hospitalization (58–61%), transport (13–22%), infections (9%–12%), administration‑related activities (11–12%) and the avoidance of catheter-related costs. Oral therapy also markedly reduced resource utilization, including 40–50 fewer hours of healthcare professional time, 8–9 fewer inpatient days, and 4–8 fewer ambulance transfers per patient. Catheter use decreased by 33%, and infection risk declined by 87% versus IV and 54% versus SC. Oral administration substantially reduces direct non‑drug costs and frees hospital capacity, offering meaningful organizational efficiencies and contributing to the sustainability of AML care within a resource‑constrained health system. These findings reinforce the value of integrating oral HMAs into clinical pathways.

## Introduction

Acute myeloid leukemia (AML) is an aggressive hematologic malignancy characterized by clonal proliferation of myeloid precursors and impaired hematopoiesis, leading to bone marrow failure and systemic complications [1]. The median age at diagnosis is approximately 68 years, and the disease predominantly affects older adults, many of whom present with comorbidities that render them unfit for intensive chemotherapy regimens [2–4]. In Spain, AML incidence continues to rise, reflecting demographic trends and improved diagnostic capabilities, thereby amplifying the need for treatment strategies that are clinically effective while minimizing hospital resource demands, respecting patients’ preferences and promoting at home treatment [5–8].

For patients deemed unsuitable for standard intensive chemotherapy, hypomethylating agents (HMA) like decitabine and azacitidine – constitute the foundation of frontline therapy [3,9]. While these agents confer meaningful clinical benefits, traditional intravenous (IV) and subcutaneous (SC) administration require frequent visits over consecutive days, prolonged chair occupancy, and substantial involvement of nursing, pharmacy, and medical staff [10,11]. These factors contribute to increased operational costs, strain on infusion capacity, and heightened infection risk, particularly in immunocompromised patients [12].

Oral formulations of HMA have emerged as a promising solution to these challenges. Oral decitabine is co‑formulated with cedazuridine, a cytidine deaminase inhibitor (DEC‑C), that increases systemic decitabine exposure through inhibition of cytidine deaminase–mediated first‑pass metabolism in the gut and liver [13]. Clinical trials have confirmed the non-inferiority of oral DEC-C in terms of efficacy and safety in patients with newly diagnosed AML who are ineligible for standard intensive chemotherapy (SIC) [14]. Beyond clinical equivalence, oral administration offers practical advantages, including reduced reliance on infusion infrastructure, lower staffing needs, and fewer hospital accesses [3,5]. These benefits align with broader healthcare objectives of improving efficiency, reducing costs, and enhancing patient autonomy and use of home-based care [5]. Existing studies in solid tumors demonstrate substantial reductions in infusion time and non‑drug costs when shifting from IV to SC delivery [10,11,15], but the incremental advantages of oral therapy in hematologic malignancies have not been fully quantified.

The present study addresses this evidence gap by evaluating the direct non‑pharmacological costs and resource utilization associated with oral DEC-C versus IV decitabine and SC azacitidine in AML patients unfit for SIC in Spain. By incorporating healthcare professional time, hospitalization needs, catheter‑related procedures, infection risk, and transport requirements, this evaluation offers a comprehensive assessment of how administration route influences the economic and organizational sustainability of AML care.

## Materials and methods

### Study design and perspective

This study was designed as a cross-sectional micro-costing analysis conducted from the perspective of the Spanish National Health System. This analysis used a bottom-up approach, whereby individual resource components were identified, measured, assessed, and subsequently aggregated to estimate total costs. The primary objective of this approach was to isolate and quantify the economic and organizational impact associated with the mode of administration of HMAs (oral versus IV and SC) in patients with AML who were considered unfit for SIC, rather than comparing pharmacological alternatives. Accordingly, drug acquisition costs were excluded, and clinical equivalence in terms of efficacy and safety was assumed based on available evidence. This methodological approach allows a focused assessment of administration-driven resource use within the Spanish National Health System.

The analysis covered a full treatment course and used a deterministic Excel‑based model that encoded all parameters, assumptions and intermediate calculations. Because the analytic horizon was less than one year, no discounting was applied.

### Analytic framework and model structure

The methodological framework was adapted from Di Costanzo et al., with modifications to reflect Spanish hospital operations and resource use patterns [16]. A bottom‑up micro‑costing approach was implemented, estimating costs across five administration‑driven domains: (i) drug preparation, dispensation and administration; (ii) treatment‑related hospitalizations; (iii) medical transportation for dosing‑day visits; (iv) catheter implantation, follow‑up and complication management; and (v) infection incidence and management. In this study, treatment‑related hospitalizations refer specifically to inpatient admissions required to complete multi‑day treatment administration, rather than admissions driven by disease progression or toxicity. Resource use was estimated per cycle and stratified into induction (cycle 1) and consolidation (cycles 2–6), with route‑specific aggregation reflecting typical Spanish practice: five cycles for IV decitabine and oral DEC‑C, aligned with the median number of treatment cycles received by patients in the pivotal oral DEC‑C clinical trial, and six for SC azacitidine, in alignment with PETHEMA recommendations on response timing for SC azacitidine [14,17].

### Data sources

The model integrated two types of data: 1) primary data, collected through a structured survey targeting healthcare professionals (HCP) directly involved in HMA delivery and supportive care in AML patients; 2) secondary data from Spanish hospital tariffs for unit costs. Together, these inputs determined route‑specific resource use per cycle and allowed estimation of whole‑treatment costs for each modality. Primary data were obtained through a structured survey conducted between March and May 2025–30 healthcare professionals across 16 hospitals in Spain. The sample included equal numbers of hematologists, nurses, and hospital pharmacists. Respondents were required to have direct responsibility for prescribing, preparing, dispensing, or administering HMAs. Respondents reported cycle-specific estimates of treatment workflows, professional time requirements, catheter-related procedures, hospitalization patterns, ambulance use, and infection management pathways. Narrative descriptions of workflows were collected to contextualize quantitative inputs and validate internal consistency. Follow-up calls were conducted to reconcile discrepancies. These items informed the domain‑specific resource quantities used in the model, which were subsequently monetized using unit costs derived from official Spanish hospital tariffs and the eSalud database for consumables, staff time, bed-days, and transport [18,19]. All unit costs are expressed in euros (€) at 2024 values and can be consulted in S1 Table.

### Assumptions and attribution rules

Model assumptions were defined a priori to isolate the effect of administration route. Cycle length was fixed at 28 days across routes; dosing‑day counts, and route‑specific cycle totals reflected Spanish practice and served only to structure aggregation of resources. For catheter use, only the subset of IV‑treated patients requiring central access for drug delivery was attributed to the administration route; catheter use in SC or oral cohorts, when present, was considered background to transfusion or other supportive‑care needs. Infection incidence was apportioned to hospital access, catheter exposure and patient‑related etiologies according to the survey and was managed in ambulatory or inpatient settings consistent with national practice. Oral administration was modeled as requiring minimal observation and no infusion chair occupancy. Hospitalization probabilities were assumed to be highest in induction and lower thereafter for all routes, according to data from the survey and consistent with published data [20]. These attribution rules were designed to ensure that differences in costs and operational burden across routes reflect administration‑driven mechanisms rather than underlying clinical heterogeneity.

### Sensitivity and scenario analyses

To assess the robustness of the base-case results to parameter uncertainty and structural assumptions, three complementary analyses were conducted.

First, a one-way deterministic sensitivity analysis (OWSA) individually varied 23 model parameters across plausible ranges while holding all other inputs at their base-case values. Ranges were derived from a prespecified hierarchy: empirical survey percentiles where available, ± 20% for tariff-sourced unit costs, ± 50% for cost items with documented regional heterogeneity, and analyst-proposed bounds for parameters lacking cycle-level survey data. The twelve most influential parameters and their tested ranges are reported in S4 Table.

Second, a probabilistic sensitivity analysis (PSA) was conducted using second-order Monte Carlo simulation with 10,000 iterations. Twenty-two uncertain parameters were simultaneously sampled from parametric distributions: Beta distributions for probabilities and proportions, calibrated to survey-derived means and standard deviations using the method of moments where feasible, and Gamma distributions for cost inputs and staff salaries. Convergence was assessed by monitoring the running mean at progressive iteration milestones (S5 Table).

Third, thirteen structural scenario analyses evaluated the impact of alternative clinical and methodological assumptions on the incremental non-drug cost (S6 Table). Scenarios addressed: (i) equalization of administration-related infection rates across routes; (ii) equalization of hospitalization probabilities, including scenarios in which all routes were assigned IV, SC, or population-weighted hospitalization rates; (iii) variation in catheter-use attribution and treatment duration; and (iv) a maximum conservative stress test combining the most unfavorable assumptions simultaneously.

Drug acquisition costs were excluded from all uncertainty analyses, consistent with the base-case study design. Results are reported in Supporting Information (S4–S6 Tables).

### Ethics statement

This study used aggregated, non-identifiable information from healthcare professionals and publicly available tariffs; no patient-level data were collected, and no ethical approval was required. Where applicable, institutional approvals for survey activities were obtained. Moreover, the study was performed according to the CHEERS (Consolidated Health Economic Evaluation Reporting Standards) practices.

## Results

### Model input parameters

Table 1 summarizes the parameterization used to quantify route‑dependent resource use. All administration pathways followed a 28‑day cycle structure. Route‑specific treatment days were 5 for oral and IV and 7 for SC, with whole‑course cycle totals of 5 (IV, oral) and 6 (SC). Days with drug preparation/dispensing – used as a proxy for hospital access – were highest for SC (7) and IV (5), and lowest for oral (1). Hospitalizations for treatment administration probabilities, derived from the HCP survey, were parameterized separately for induction (cycle 1: IV 50%, SC 40%, oral 20%) and consolidation (cycles 2 + : IV 30%, SC 20%, oral 10%). HCP reported that most patients received HMA through SC route (73%), followed by IV (20%) and oral (7%). The probability of having a central venous catheter was higher for patients on IV treatment (77%) than for those on SC (44%) or oral (23%) alternatives. For the model, we assumed that the difference in catheter use between SC users – who account for most of AML unfit patients – and the IV ones (33%) could be attributed to the IV administration route. HCP reported that 44% of patients developed a moderate or severe infection during treatment, of which 21% from the catheter (9% weighted incidence), 35% from visits to the hospital (16% weighted incidence), 22% related to other pathologies, and 22% from other causes. In the model, infection risk was decomposed into catheter‑related and hospital‑access–related components, scaled by route‑specific visit frequency and use of catheter attributable to the administration route. Over the full course, weighted treatment‑related infection risks were IV 25%, SC 22%, and oral 3%.

**Table 1:** Model input parameters.

Domain	Variable (definition/unit)	IV	SC	Oral	Notes
Cycle framework	Cycle length (days)	28	28	28	With route‑specific cycle counts.
	Treatment days per cycle (dosing days)	5	7	5	Dosing schedule per route.
	Days with drug preparation and/or dispensing per cycle	5	7	1	Used as proxy of hospital access per month per treatment.
	Total cycles (whole course)	5	6	5	Reflects Spanish practice.
Current treatment	Patients on HMA treatment per route (%)	20	73	7	From HCP survey.
Catheter due to administration route	Patients with catheter (%)	77	44	23	From HCP survey.
	Patients with catheter attributable to route (%)	33	0	0	Model assumption from catheters use per route from HCP survey.
	Complication, risk of thrombosis rate after implant (%)	9			From HCP survey. Applied to catheter users only.
	Catheter routine follow‑up visits per cycle (n)	4			
	Extra nurse time to manage thrombosis (min)	37			From HCP survey. Per Thrombosis episode.
	Extra hematologist time (min)	31			
	Post‑infection catheter events, re‑implant after catheter‑related infection (%)	40			From HCP survey. Applied where infection occurs.
	Post‑infection catheter events, extraction after catheter‑related infection (%)	26			
Incidence of infections	Incidence of infections over treatment course caused by central catheter	9			From HCP survey. Applied to patients with catheters due to administration route.
	Incidence of infections over treatment course caused by visits to the hospital	16			From HCP survey. Applied to patients based on their number of visits to the hospital for treatment.
	Overall weighted risk of treatment related infection	25	22	3	Computed considering the number of hospital visits and catheter use.
Hospitalization for treatment‑related observation	Admission share, Cycle 1 (%)	50	40	20	From HCP survey, when provided.
	Admission share, Cycles 2+ (%)	30	20	10	
Ambulance use	Proportion of patients requiring ambulance for dosing‑day visits (%)	28			From HCP survey. Scaled by dosing‑day and admission status.

HCP, healthcare professionals. HMA, hypomethylating agents. IV, Intravenous. SC, Subcutaneous.

### Healthcare professionals time

Table 2 summarizes the total minutes dedicated by HCP for activities related to catheter management and drug preparation, dispensing and/or administration, and the respective estimated cost. The detailed time reported by HCP in the survey per sub-activity and segregated by HCP type is presented in S2 and S3 Tables.

**Table 2:** Healthcare professionals’ time and cost per activity, patient and cycle.

Broad activity	Patient Type	Cycle	Minutes (euros) per patient and cycle		
			IV	SC	Oral
Catheter management	Patients with catheter	Cycle 1	204 min. (€55)	–	–
	Patients with catheter	Cycle 2+	138 min. (€34)	–	–
Drug preparation, dispensing and/or administration (per day)	Patients with catheter	Cycle 1	119 min. (€38)	74 min. (€25)	12 min. (€5)
	Patients with catheter	Cycle 2+	110 min. (€35)	72 min. (€24)	12 min. (€4)
	Patients without catheter	Cycle 1	93 min. (€29)	74 min. (€25)	12 min. (€5)
	Patients without catheter	Cycle 2+	93 min. (€29)	72 min. (€24)	12 min. (€4)

IV, Intravenous. SC, Subcutaneous.

### Whole-treatment non-drug costs

Across the entire treatment course, non‑drug direct costs per patient were €9,998 for IV, €12,177 for SC, and €1,306 for oral administration (Table 3). These totals were composed of drug administration costs of €957, €1,323, and €23 respectively; treatment‑related hospitalization of €6,042, €6,966, and €711; ambulance transport of €1,521, €2,808, and €418; infection management of €1,220, €1,080, and €154; and catheter management attributable to IV route of €257. In cycle 1, non‑drug totals were €2,564 (IV), €2,781 (SC), and €831 (oral). Hospitalization dominates parenteral costs in cycle 1 because of higher admission shares and multi‑day dosing. For cycles 2 + , per‑cycle non‑drug totals were €1,858 (IV), €1,879 (SC), and €119 (oral), reflecting persistent multi‑day dosing for parenteral delivery and minimal on‑site needs for oral dispensation.

**Table 3:** Estimated cost per patient per administration modality, type of cost and cycle.

Type of cost	IV	SC	Oral	Oral vs IV (%)	Oral vs SC (%)
**Full treatment**	**9,998 €**	**12,177 €**	**1,306 €**	**−87%**	**−89%**
Drug administration	957 €	1,323 €	23 €	−98%	−98%
Hospitalizations	6,042 €	6,966 €	711 €	−88%	−90%
Ambulance	1,521 €	2,808 €	418 €	−73%	−85%
Catheter	257 €	0 €	0 €	−100%	0%
Infections	1,220 €	1,080 €	154 €	−87%	−86%
**Cycle 1**	**2,564 €**	**2,781 €**	**831 €**	**−68%**	**−70%**
Drug administration	196 €	226 €	5 €	−97%	−98%
Hospitalizations	1,777 €	1,990 €	711 €	−60%	−64%
Ambulance	251 €	384 €	84 €	−67%	−78%
Catheter	97 €	0 €	0 €	−100%	0%
Infections	244 €	180 €	31 €	−87%	−83%
**Cycle 2+**	**1,858 €**	**1,879 €**	**119 €**	**−94%**	**−94%**
Drug administration	190 €	219 €	4 €	−98%	−98%
Hospitalizations	1,066 €	995 €	0 €	−100%	−100%
Ambulance	318 €	485 €	84 €	−74%	−83%
Catheter	40 €	0 €	0 €	−100%	0%
Infections	244 €	180 €	31 €	−87%	−83%

IV, Intravenous. SC, Subcutaneous. Vs, versus.

### Operational and organizational outcomes

Route‑attributable professional minutes required for drug administration were lower for oral therapy as shown in Table 4. Over the full course, IV and SC administration required approximately 2,476 and 3,040 minutes per patient, respectively, compared with 62 minutes for oral DEC‑C. When catheter‑management time was included, IV rose by roughly 248 minutes to a combined 2,724 minutes, whereas SC and oral remained essentially unchanged. Treatment‑related inpatient days were approximately nine for IV and ten for SC, compared with about one day under the oral route. Infection‑related hospital days added roughly two to three days for parenteral routes and were near zero for oral therapy, yielding about eleven to twelve total route‑attributable hospital days for IV/SC and one day for oral. Ambulance transports followed the dose‑day structure and admission patterns: when aggregated over the entire treatment course and expressed per patient, the model estimated a mean of approximately five round‑trip ambulance transports for IV therapy, nine for SC therapy, and between one and one point four for oral therapy, reflecting the substantially lower need for facility‑based administration with oral treatment.

**Table 4:** Operational resource use per patient (whole treatment course).

Outcome	IV	SC	Oral	Oral vs IV (%)	Oral vs SC (%)
HCP time (minutes) for drug administration	2,476	3,040	62	−97%	−98%
Inpatient days	9	10	1	−88%	−90%
Ambulance transports	5	9	1	−73%	−85%
HCP time (minutes) for catheter management	248	–	–	−100%	0%
Infections additional inpatient days	3	2	0	−87%	−86%

HCP, Healthcare professionals. IV, Intravenous. SC, Subcutaneous. Vs, versus. Minutes are route‑attributed professional minutes; days are inpatient bed‑days. All values in this table are from the base model.

### Sensitivity and scenario analyses

Full results of the sensitivity and scenario analyses are presented in Supporting Information (S4–S6 Tables).

Oral DEC‑C remained less costly than both IV decitabine and SC azacitidine in all 46 one-way parameter variations tested in the one-way deterministic sensitivity analysis (S4 Table). The most influential parameters for the oral-versus-IV comparison were the consolidation-cycle hospitalization probability for IV therapy, the consolidation-cycle hospitalization probability for oral therapy, and the inpatient day cost. Even under the single least favorable parameter value, oral therapy retained savings of at least €5,849 versus IV (−67% of the base case) and €8,028 versus SC (−74%).

Across 10,000 Monte Carlo iterations, the mean (95% credible interval) incremental saving with oral therapy was €7,280 (€3,382–€12,532) versus IV and €9,442 (€4,152–€16,436) versus SC (S5 Table). The probability that oral DEC‑C had lower non-drug costs was 100.0% versus IV and 99.99% versus SC.

Oral DEC‑C remained the least costly administration route in all 13 structural scenarios (S6 Table). When hospitalization rates were equalized at IV levels, oral therapy still saved €3,361 versus IV (−34%) and €8,225 versus SC (−55%). Under the maximum conservative stress test, which simultaneously equalized hospitalization rates, removed administration-related infection differentials, reduced catheter attribution to its lower bound, and harmonized treatment duration, oral therapy retained non-drug savings of €2,116 versus IV (−25%) and €5,153 versus SC (−44%).

## Discussion

This study shows that the route of administration is a major determinant of hospital expenditure and operational burden in AML patients unfit for intensive chemotherapy in Spain. Across the full treatment course, oral administration reduced total non‑drug direct costs by nearly 90% compared with IV and SC routes (€1,306 versus €9,998 and €12,177), mainly driven by lower rates of hospitalization for treatment administration, elimination of multi‑day infusion workflows and catheter‑related procedures, and reduced ambulance use. These findings reflect structural efficiencies of oral therapy, which removes the need for infusion chairs, pharmacy compounding, and nurse‑led administration, enabling substantial redeployment of healthcare professionals’ time.

Our results align with international evidence, which also shows that switching from parenteral to non‑IV formulations reduces resource use in oncology treatments [10,11,15]. A related micro-costing study in Italy estimated a 78% reduction in non‑drug costs with oral DEC‑C versus IV decitabine [16]. Similarly, Spanish studies have shown that SC azacitidine is more cost‑efficient than IV delivery [21]; however, our study indicates that oral administration provides even greater organizational gains.

In our model, total HCP time for drug administration decreased by over 97% with oral, from 2,476 minutes in IV and 3,040 minutes in SC to just 62 minutes with oral therapy, reflecting the elimination of infusion‑related workflows. These results are consistent with Di Costanzo et al. versus IV, since a 93.1% reduction was reported in administration costs (€2,448.2 versus €169.2), while our study shows a 98% reduction (€957 versus €23), supporting a similar magnitude of impact of the administration route [16]. Furthermore, SC and oral formulations facilitate at-home treatment, aligning with post-pandemic strategies to minimize hospital exposure [5,22].

Reduced infection burden contributes meaningfully to the observed savings. The infection rates included in our model refer to infections linked to procedural and care‑delivery factors, rather than treatment-related adverse events driven by pharmacokinetics or intrinsic drug safety profiles. Lower exposure to hospital environments and the absence of central venous catheters resulted in considerably fewer infections and near‑zero infection‑related hospital days. In our study, infection-related costs per patient fell from €1,200 (IV) and €1,080 (SC) to just €154 with oral, a reduction of 87% and 86%, respectively. This is consistent with evidence showing elevated infection risk among immunocompromised patients with central lines [12].

Importantly, oral and IV decitabine have demonstrated pharmacokinetic and clinical equivalence, allowing patient preferences and economic and organizational considerations to inform route selection when outcomes are comparable [14]. Patient‑reported data supports the use of oral administration, with convenience, autonomy, and fewer hospital visits cited as key advantages [7,8]. Illustratively, in a multinational study, 76% of AML patients expressed a preference for oral over injectable treatments when efficacy and safety were equivalent [7]. From a hospital perspective, transitioning eligible patients to oral DEC‑C can ease pressure on day‑hospital capacity, free nursing and pharmacy time, and reduce transport coordination needs. For patients, fewer visits and avoidance of invasive procedures may support autonomy and reduce exposure to hospital‑acquired infections. Together, these benefits support integrating oral DEC‑C into Spanish AML care pathways as an efficient, effective, and patient‑centred strategy.

A key contribution of this study is the incorporation of differences in treatment‑administration–related hospitalizations across routes, informed directly by the HCP survey. Previous economic analyses did not account for these administration‑driven admissions, despite evidence suggesting that a substantial proportion of hospitalizations in this population are linked to treatment administration itself [20]. Although the inclusion of broader cost components such as hospitalization and transportation may limit direct comparability with other studies, these elements were deliberately incorporated to reflect the clinical and organizational implications of treatment administration in real-world practice. In patients with AML who are unfit for intensive chemotherapy, the route of administration can influence the frequency of hospital visits, the need for invasive procedures, and the risk of complications, all of which are closely linked to healthcare resource utilization. Therefore, these factors were considered clinically relevant in the context of this analysis. However, real‑world data on the underlying reasons for these admissions, and the degree to which they may be modifiable by route of administration, remain limited and should be investigated further. One-way deterministic, probabilistic (10,000 Monte Carlo iterations), and scenario analyses confirmed that the non-drug cost advantage of oral DEC‑C is robust to plausible variation in all tested parameters and structural assumptions. Hospitalization probability was identified as the most influential parameter, however, even when hospitalization rates were fully equalized across routes, oral therapy retained meaningful savings driven by structural differences in administration workflows, transport, and infection exposure. The magnitude of the saving ranged from approximately €2,000 to €9,000 versus IV depending on the assumptions adopted.

Nevertheless, some limitations warrant consideration. The model relied on survey‑based estimates rather than direct observational data, and the sample may not fully capture practice variations across Spanish hospitals. Although the number of experts included may be considered a limitation, the panel was intentionally designed to ensure representation across diverse Spanish autonomous communities and healthcare settings, including high-expertise leading centres and regions with more heterogeneous clinical practice, thereby enhancing the robustness and transferability of the resource use estimates. Assumptions regarding catheter attribution and hospitalization needs, particularly for oral therapy where real‑world experience remains limited, should be validated with prospective data and could impact the generalizability of the findings. Future research incorporating time‑and‑motion studies, prospective hospitalization data for the oral route, and societal costs would strengthen the evidence base. Finally, these findings should be interpreted within the study scope, which focuses on administration-related non-drug costs. Drug acquisition costs, which are subject to confidential hospital-level pricing agreements in Spain and may vary substantially across institutions, were excluded by design and could influence the total-cost comparison, particularly versus SC azacitidine. Future institution-specific analyses incorporating actual procurement costs would provide a more comprehensive economic assessment.

## Conclusions

Overall, the findings support integrating oral DEC‑C into Spanish AML care pathways for patients unfit for intensive chemotherapy. For hospitals, transitioning eligible patients to oral therapy can alleviate pressure on infusion capacity, reduce inpatient monitoring needs, and decrease reliance on medical transport. For patients, fewer hospital visits and avoidance of invasive procedures improve autonomy and safety. Taken together, these benefits position oral DEC‑C as an efficient, effective, and patient‑centered strategy that enhances the sustainability of AML care in Spain.

## Supporting information

S1 Table Unit cost. (DOCX)

S2 Table Healthcare professionals estimated time per activity related to catheter management, by administration modality (minutes). (DOCX)

S3 Table Healthcare professionals estimated time per activity related to drug administration, by administration modality and cycle (minutes). (DOCX)

S4 Table One-way deterministic sensitivity analysis results. (DOCX)

S5 Table Summary statistics from the probabilistic sensitivity analysis. (DOCX)

S6 Table Scenario analysis: non-drug costs per patient under alternative structural assumptions. (DOCX)
