# Supplementary material for: Economic and organizational impact of oral versus intravenous and subcutaneous administration of hypomethylating agents in patients with acute myeloid leukemia not eligible for intensive chemotherapy in Spain
Source: PLoS One. 2026 Sep 22;21(9):e0358426. doi: 10.1371/journal.pone.0358426 (PMC13596810; doi:10.1371/journal.pone.0358426)
Supplement: Supplementary file 1 [file pone.0358426.s001.docx]

**Table S1. Unit cost**

| **Cost** | **Value (€)** |
| --- | --- |
| Mean cost per inpatient day (DRG 695) | € 711 |
| Mean cost per hospitalization (DRG 695) | € 2,341 |
| Mean cost per episode of infection managed at outpatient setting | € 533 |
| Mean incremental cost of hospitalizations with an associated hospital acquired infection | € 9,349 |
| Mean cost per ambulance transportation (round-trip) | € 300 |
