# Supplementary material for: Economic and organizational impact of oral versus intravenous and subcutaneous administration of hypomethylating agents in patients with acute myeloid leukemia not eligible for intensive chemotherapy in Spain
Source: PLoS One. 2026 Sep 22;21(9):e0358426. doi: 10.1371/journal.pone.0358426 (PMC13596810; doi:10.1371/journal.pone.0358426)
Supplement: Supplementary file 2 [file pone.0358426.s002.docx]

**Table S2. Healthcare professionals estimated time per activity related to catheter management, by administration modality (minutes)**

| **Item** | **Healthcare personnel** | **IV** | **SC** | **Oral** |
| --- | --- | --- | --- | --- |
| Catheter insertion | Nurse | 52.27 | 52.27 | 52.27 |
| Catheter insertion | Hematologist | 23.74 | 23.74 | 23.74 |
| Catheter insertion | Laboratory technician | 0.00 | 0.00 | 0.00 |
| Catheter insertion | Pharmacist | 0.00 | 0.00 | 0.00 |
| Catheter insertion | Administrative staff | 0.00 | 0.00 | 0.00 |
| Catheter insertion | Other healthcare worker | 26.00 | 26.00 | 26.00 |
| Catheter follow‑up | Nurse | 21.00 | 21.00 | 21.00 |
| Catheter follow‑up | Hematologist | 0.00 | 0.00 | 0.00 |
| Catheter follow‑up | Laboratory technician | 0.00 | 0.00 | 0.00 |
| Catheter follow‑up | Pharmacist | 0.00 | 0.00 | 0.00 |
| Catheter follow‑up | Administrative staff | 0.00 | 0.00 | 0.00 |
| Catheter follow‑up | Other healthcare worker | 13.00 | 13.00 | 13.00 |
| Catheter removal | Nurse | 27.00 | 27.00 | 27.00 |
| Catheter removal | Hematologist | 0.00 | 0.00 | 0.00 |
| Catheter removal | Laboratory technician | 0.00 | 0.00 | 0.00 |
| Catheter removal | Pharmacist | 0.00 | 0.00 | 0.00 |
| Catheter removal | Administrative staff | 0.00 | 0.00 | 0.00 |
| Catheter removal | Other healthcare worker | 29.00 | 29.00 | 29.00 |

IV, intravenous. SC, subcutaneous.
