# Supplementary material for: Economic and organizational impact of oral versus intravenous and subcutaneous administration of hypomethylating agents in patients with acute myeloid leukemia not eligible for intensive chemotherapy in Spain
Source: PLoS One. 2026 Sep 22;21(9):e0358426. doi: 10.1371/journal.pone.0358426 (PMC13596810; doi:10.1371/journal.pone.0358426)
Supplement: Supplementary file 3 [file pone.0358426.s003.docx]

**Table S3. Healthcare professionals estimated time per activity related to drug administration, by administration modality and cycle (minutes)**

| **Item** | **Healthcare personnel** | **Cycle 1** | | | **Cycle 2+** | | |
| --- | --- | --- | --- | --- | --- | --- | --- |
|  |  | **IV** | **SC** | **Oral** | **IV** | **SC** | **Oral** |
| Drug preparation | Nurse | 11.67 | 10.00 | 0.00 | 11.67 | 10.00 | 0.00 |
| Drug preparation | Hematologist | 4.90 | 4.90 | 0.00 | 4.90 | 4.90 | 0.00 |
| Drug preparation | Laboratory technician | 12.83 | 8.22 | 0.00 | 12.83 | 8.22 | 0.00 |
| Drug preparation | Pharmacist | 18.33 | 16.50 | 0.00 | 18.33 | 16.50 | 0.00 |
| Drug preparation | Administrative staff | 0.00 | 0.00 | 0.00 | 0.00 | 0.00 | 0.00 |
| Drug preparation | Other healthcare worker | 15.59 | 15.86 | 0.00 | 15.59 | 15.86 | 0.00 |
| Drug dispensation | Nurse | 0.00 | 0.00 | 0.66 | 0.00 | 0.00 | 1.06 |
| Drug dispensation | Hematologist | 0.00 | 0.00 | 4.88 | 0.00 | 0.00 | 3.45 |
| Drug dispensation | Laboratory technician | 0.00 | 0.00 | 0.00 | 0.00 | 0.00 | 0.00 |
| Drug dispensation | Pharmacist | 0.00 | 0.00 | 5.50 | 0.00 | 0.00 | 2.98 |
| Drug dispensation | Administrative staff | 0.00 | 0.00 | 1.25 | 0.00 | 0.00 | 4.96 |
| Drug dispensation | Other healthcare worker | 0.00 | 0.00 | 0.00 | 0.00 | 0.00 | 0.00 |
| Drug administration, in patients with catheter | Nurse | 48.50 | 11.80 | 0.00 | 41.00 | 10.80 | 0.00 |
| Drug administration, with catheter | Hematologist | 4.18 | 4.38 | 0.00 | 3.30 | 3.50 | 0.00 |
| Drug administration, with catheter | Laboratory technician | 0.00 | 0.00 | 0.00 | 0.00 | 0.00 | 0.00 |
| Drug administration, with catheter | Pharmacist | 3.00 | 2.70 | 0.00 | 2.52 | 2.22 | 0.00 |
| Drug administration, with catheter | Administrative staff | 0.00 | 0.00 | 0.00 | 0.00 | 0.00 | 0.00 |
| Drug administration, with catheter | Other healthcare worker | 0.00 | 0.00 | 0.00 | 0.00 | 0.00 | 0.00 |
| Drug administration, without catheter | Nurse | 28.50 | 11.80 | 0.00 | 28.50 | 10.80 | 0.00 |
| Drug administration, without catheter | Hematologist | 0.25 | 4.38 | 0.00 | 0.13 | 3.50 | 0.00 |
| Drug administration, without catheter | Laboratory technician | 0.00 | 0.00 | 0.00 | 0.00 | 0.00 | 0.00 |
| Drug administration, without catheter | Pharmacist | 1.11 | 2.70 | 0.00 | 0.67 | 2.22 | 0.00 |
| Drug administration, without catheter | Administrative staff | 0.00 | 0.00 | 0.00 | 0.00 | 0.00 | 0.00 |
| Drug administration, without catheter | Other healthcare worker | 0.00 | 0.00 | 0.00 | 0.00 | 0.00 | 0.00 |

IV, intravenous. SC, subcutaneous.
