# Supplementary material for: Economic and organizational impact of oral versus intravenous and subcutaneous administration of hypomethylating agents in patients with acute myeloid leukemia not eligible for intensive chemotherapy in Spain
Source: PLoS One. 2026 Sep 22;21(9):e0358426. doi: 10.1371/journal.pone.0358426 (PMC13596810; doi:10.1371/journal.pone.0358426)
Supplement: Supplementary file 4 [file pone.0358426.s004.docx]

**Table S4. One-way deterministic sensitivity analysis results**

| **Parameter** | **Parameters scenarios** | | | **Δ Oral - IV (€/patient)** | | **Δ Oral - SC (€/patient)** | |
| --- | --- | --- | --- | --- | --- | --- | --- |
|  | **Base** | **Low** | **High** | **Low** | **High** | **Low** | **High** |
| Hospitalization probability, consolidation cycles – IV | 0.30 | 0.15 | 0.45 | −6,760 | −10,624 | −10,872 | −10,872 |
| Hospitalization probability, consolidation cycles – oral | 0.00 | 0.00 | 0.20 | −8,692 | −5,849 | −10,872 | −8,028 |
| Cost per inpatient day (DRG 695) | 710.8 | 568.7 | 853.0 | −7,626 | −9,759 | −9,621 | −12,123 |
| Ambulance use probability | 0.279 | 0.05 | 0.55 | −7,787 | −9,767 | −8,910 | −13,200 |
| Overall infection incidence | 0.441 | 0.20 | 0.70 | −8,110 | −9,318 | −10,366 | −11,415 |
| Cost of hospital-acquired infection | 9,349 | 4,675 | 14,024 | −8,188 | −9,197 | −10,434 | −11,309 |
| Hospitalization probability, induction cycle – IV | 0.50 | 0.35 | 0.65 | −8,209 | −9,175 | −10,872 | −10,872 |
| Hospital-access infection attribution | 0.354 | 0.025 | 0.575 | −8,119 | −9,078 | −10,011 | −11,449 |
| Cost per ambulance round-trip | 300 | 200 | 450 | −8,325 | −9,244 | −10,075 | −12,067 |
| Catheter-related infection attribution | 0.206 | 0.05 | 0.40 | −8,352 | −9,115 | −10,872 | −10,872 |
| Infection management, ambulatory share | 0.50 | 0.30 | 0.70 | −9,073 | −8,312 | −11,202 | −10,541 |
| Hospitalization probability, induction cycle – oral | 0.20 | 0.10 | 0.30 | −9,048 | −8,337 | −11,227 | −10,516 |

IV, intravenous. SC, subcutaneous.

Δ = incremental non-drug cost (oral minus comparator, € per patient); negative values indicate oral is less costly. Base-case: oral vs IV = − €8,692; oral vs SC = − €10,872. Oral DEC‑C was less costly than both comparators in all 46 one-way tests (23 parameters × 2 extremes). Results are shown for the twelve most influential parameters, ranked by variation on the oral-versus-IV incremental non-drug cost in comparison to the base case. Remaining 11 parameters (variation versus base case inferior to €700) are available upon request.
