# Supplementary material for: Economic and organizational impact of oral versus intravenous and subcutaneous administration of hypomethylating agents in patients with acute myeloid leukemia not eligible for intensive chemotherapy in Spain
Source: PLoS One. 2026 Sep 22;21(9):e0358426. doi: 10.1371/journal.pone.0358426 (PMC13596810; doi:10.1371/journal.pone.0358426)
Supplement: Supplementary file 5 [file pone.0358426.s005.docx]

**Table S5. Summary statistics from the probabilistic sensitivity analysis**

| **Outcome** | **Mean** | **Median** | **SD** | **P2.5** | **P97.5** |
| --- | --- | --- | --- | --- | --- |
| IV total non-drug cost | €10,009 | €9,707 | €2,610 | €5,772 | €15,965 |
| SC total non-drug cost | €12,170 | €11,803 | €3,534 | €6,510 | €20,008 |
| Oral total non-drug cost | €2,728 | €2,608 | €960 | €1,221 | €4,958 |
| Incremental cost, oral vs IV | −€7,280 | −€7,069 | €2,336 | −€12,532 | −€3,382 |
| Incremental cost, oral vs SC | −€9,442 | −€9,130 | €3,203 | −€16,436 | −€4,152 |

IV, intravenous. SC, subcutaneous. SD, standard deviation.

Probability that oral non-drug cost is inferior to IV = 100.0% (10,000/10,000 iterations). Probability that oral non-drug cost is inferior to SC = 99.99% (9,999/10,000). P2.5 and P97.5 define the 95% credible interval. The probabilistic sensitivity analysis mean oral cost (€2,728) exceeds the deterministic base case (€1,306) because the probabilistic sensitivity analysis centers the consolidation hospitalization probability for oral therapy at 10% rather than 0% (deterministic floor).
