# Supplementary material for: Economic and organizational impact of oral versus intravenous and subcutaneous administration of hypomethylating agents in patients with acute myeloid leukemia not eligible for intensive chemotherapy in Spain
Source: PLoS One. 2026 Sep 22;21(9):e0358426. doi: 10.1371/journal.pone.0358426 (PMC13596810; doi:10.1371/journal.pone.0358426)
Supplement: Supplementary file 6 [file pone.0358426.s006.docx]

**Table S6. Scenario analysis: non-drug costs per patient under alternative structural assumptions**

| **Scenario** | **Description** | **€/patient** | | | **Δ Oral - IV** | | **Δ Oral - SC** | |  |
| --- | --- | --- | --- | --- | --- | --- | --- | --- | --- |
|  |  | **IV** | **SC** | **Oral** | **€/patient** | **% IV** | **€/patient** | **% SC** | |
| Base case | — | €9,998 | €12,177 | €1,306 | −€8,692 | −87% | −€10,872 | −89% | |
| Equalized administration-related infection | Catheter- and hospital-access infection attribution set to zero for all routes | €8,778 | €11,097 | €1,151 | −€7,626 | −87% | −€9,946 | −90% | |
| Partially equalized infection | Catheter-related mechanism retained for IV but hospital-access attribution removed | €9,226 | €11,097 | €1,151 | −€8,075 | −88% | −€9,946 | −90% | |
| Higher oral hospitalization rates | Oral admission probabilities increased to 35% (Cycle 1) and 20% (Cycles 2+) | €9,998 | €12,177 | €4,682 | −€5,316 | −53% | −€7,495 | −62% | |
| Harmonized treatment duration | All routes set to 5 treatment cycles | €9,998 | €10,478 | €1,306 | −€8,692 | −87% | −€9,172 | −88% | |
| Upper-bound catheter attribution | IV-attributable catheter proportion increased to 50% | €10,131 | €12,177 | €1,306 | −€8,826 | −87% | −€10,872 | −89% | |
| Lower-bound catheter attribution | IV-attributable catheter proportion decreased to 10% | €9,819 | €12,177 | €1,306 | −€8,513 | −87% | −€10,872 | −89% | |
| Combined conservative assumptions | Equalized infection + higher oral hospitalization rate + lower catheter + harmonized duration | €8,599 | €9,398 | €4,528 | −€4,071 | −47% | −€4,870 | −52% | |
| Equalized hospitalization at IV rates | All routes: Cycle 1=50%, Cycles 2+=30% | €9,998 | €14,862 | €6,637 | −€3,361 | −34% | −€8,225 | −55% | |
| Equalized hospitalization at SC rates | All routes: Cycle 1=40%, Cycles 2+=20% | €8,388 | €12,177 | €4,860 | −€3,528 | −42% | −€7,317 | −60% | |
| Equalized hospitalization at weighted-average rates | All routes: Cycle 1=40.6%, Cycles 2+=20.6% | €8,484 | €12,338 | €4,966 | −€3,518 | −41% | −€7,372 | −60% | |
| Hospitalization costs excluded | Hospitalization removed for all routes | €4,524 | €5,913 | €595 | −€3,929 | −87% | −€5,318 | −90% | |
| Maximum conservative stress test | Equalized hosp. (IV rates) + equalized infection + lower catheter + harmonized duration | €8,599 | €11,635 | €6,483 | −€2,116 | −25% | −€5,153 | −44% | |

IV, intravenous. SC, subcutaneous.

Drug acquisition costs excluded. Δ = incremental non-drug cost (oral minus comparator, € per patient); negative values indicate oral is less costly. Base-case: oral vs IV = − €8,692; oral vs SC = − €10,872. Oral DEC‑C was less costly than both comparators in all 46 one-way tests (23 parameters × 2 extremes). Cycle 1 (induction); Cycles 2+ (consolidation). Results are shown for the twelve most influential parameters, ranked by variation on the oral-versus-IV incremental non-drug cost in comparison to the base case. Remaining 11 parameters (variation versus base case inferior to €700) are available upon request.
